# Longitudinal White Matter Diffusion Alterations in Medication Overuse Headache Before and After Medication Withdrawal: A Diffusion Tensor Imaging Study

**DOI:** 10.3390/medicina62101906

**Published:** 2026-10-02

**Authors:** Murat Polat, Gozde Kizilates-Evin, Kerim Aslan, Huseyin Alparslan Sahin, Taner Ozbenli

**Affiliations:** 1 Department of Neurology, Faculty of Medicine, Ondokuz Mayıs University, 55139 Samsun, Türkiye; hasahin@omu.edu.tr; 2 Hulusi Behçet Life Sciences Research Laboratory, Neuroimaging Unit, Istanbul University, 34093 Istanbul, Türkiye; gozde.kizilates@gmail.com; 3 Department of Neuroradiology, Faculty of Medicine, Ondokuz Mayıs University, 55139 Samsun, Türkiye; mdkerim@hotmail.com; 4 Independent Researcher, 55200 Samsun, Türkiye; tanerozben@gmail.com

**Keywords:** medication overuse headache, corpus callosum, diffusion tensor imaging, tract-based spatial statistics, white matter, fractional anisotropy

## Abstract

*Background and Objectives*: The pathophysiology of medication overuse headache (MOH) remains incompletely understood, and advanced neuroimaging methods such as Diffusion Tensor Imaging (DTI) may help clarify the structural white matter (WM) changes associated with chronic analgesic overuse. The objective of this study was to identify WM microstructural alterations in patients with MOH, both before and after discontinuation of the overused medication, compared with healthy controls (HC), utilizing whole-brain and region-of-interest (ROI)-restricted tract-based spatial statistics (TBSS). *Materials and Methods*: Twenty-three patients with MOH, diagnosed according to ICHD (3rd edition) criteria, and 23 demographically matched HC underwent 3-Tesla DTI. Whole-brain and ROI-restricted TBSS analyses of fractional anisotropy (FA), axial diffusivity (AD), mean diffusivity (MD) and radial diffusivity (RD) were performed to assess global and tract-specific white matter microstructural alterations. In the MOH group, DTI was repeated at follow-up after withdrawal of the overused medication, initiation of alternative treatment, and clinical improvement (MOH-DW). Sixteen patients and 16 controls were included in the final analysis after exclusions for pregnancy, loss to follow-up, or technical issues. *Results*: Whole-brain TBSS revealed no statistically significant group differences. ROI-restricted analysis revealed a marked increase in fractional anisotropy (FA) and axial diffusivity (AD) in the MOH group relative to the HC group within the left anterior limb of the internal capsule, the body of the corpus callosum, and the right superior corona radiata. Concurrently, higher mean diffusivity (MD) was observed in the left anterior limb of the internal capsule. At the follow-up assessment, FA and AD remained significantly higher than in healthy controls in the anterior limb of the internal capsule and superior corona radiata, whereas the group difference in the corpus callosum was no longer statistically significant. As the direct within-subject comparison between pre- and post-withdrawal measurements did not reach significance in any region, this may reflect the loss of a detectable group difference rather than confirmed within-subject reversibility. *Conclusions*: MOH may be associated with WM microstructural changes characterized predominantly by increased, rather than decreased, diffusion anisotropy and axial diffusivity in tracts relevant to pain processing. The disappearance of the significant group difference in the corpus callosum at follow-up raises the hypothesis that this may be compatible with a dynamic and potentially reversible microstructural process; however, because medication withdrawal occurred concurrently with the introduction of alternative treatment, this finding cannot be attributed specifically to withdrawal. Given the modest sample size and the absence of correction across the 27 ROI analyses tested, these exploratory findings are preliminary and warrant confirmation in larger, independent longitudinal cohorts.

## 1. Introduction

Medication overuse headache (MOH) can be described as a headache condition that develops as a result of the regular, excessive use of acute headache medications for more than three months, presenting on 15 or more days per month [1]. MOH is not the primary type of chronic daily headache but often accompanies primary headaches and other secondary headaches [2]. MOH has been reported most frequently in cases of migraine and tension-type headache [3,4]. MOH is one of the top 20 causes of disability in the world [2]. The available literature predominantly suggests that MOH affects approximately 1–2% of the general population, with a notably greater prevalence among women compared to men [5,6,7,8]. The prevalence of MOH is estimated to be nearly 50% among patients referred to specialised headache centres [9]. The pathophysiology of MOH remains to be fully elucidated. However, it is hypothesised that genetic and biobehavioral factors, as well as central and peripheral sensitization, cortical spreading depression, changes in neurotransmitter levels, and activation of the trigeminovascular system may play a role [9,10,11].

A systematic review reported that 33 genes involving 50 polymorphisms were found to be associated with an increased susceptibility to MOH [12]. Investigations have indicated that central sensitization, the same process that occurs in migraine, may contribute to the development of MOH in patients with chronic headache. This is due to the facilitation of trigeminal pain processing [5,6,13]. It is hypothesized that chronic exposure to triptans and other analgesics in humans may result in downregulation of serotonin receptors and alterations in central inhibitory pathways, resulting in impaired antinociceptive activity and a persistent headache [11]. It is important to note that this effect is not limited to triptans alone, but also includes other analgesics. Neurophysiological research has lent support to this notion by revealing enhanced facilitation of trigeminal and somatic nociceptive pathways in MOH, a process thought to occur predominantly at the supraspinal level [14]. Drug-related mechanisms may also contribute to headache chronification. Dopaminergic circuits implicated in addictive behaviour are thought to play a role: the prefrontal cortex and ventral striatum receive dopaminergic input and participate in drug-induced central sensitization, while the dorsal striatum, innervated from the substantia nigra, is linked to the persistence of medication-overuse habits [15]. The interaction between the prefrontal cortex and limbic learning circuits is also considered central to the transition from acute to chronic pain, which has been conceptualized as a state of persistent learning in which the constant presence of pain prevents extinction [16]. As nociceptive input intensifies, limbic structures including the nucleus accumbens, amygdala, and hippocampus are increasingly engaged, giving rise to maladaptive learned associations [17]. MOH has been proposed as a bio-behavioral disorder. Behaviors that can lead to the overuse of medication include fear of headaches, drug-taking behavior, anticipatory anxiety, and psychological drug dependence [18].

Although the disease is diagnosed clinically, it has been demonstrated that advanced neuroimaging methods, including Diffusion Tensor Imaging (DTI), can provide supplementary information. DTI can detect abnormalities even when routine MRI is normal. DTI data may be examined using the Tract-Based Spatial Statistics (TBSS) approach, enabling the assessment of diffusivity across all spatial directions. This approach can uncover subtle variations in white matter (WM) microstructure through comparative analysis of group-level data. In chronic pain conditions, structural neuroimaging techniques have provided valuable information. Studies suggest that long-term exposure to pain can cause structural changes in several brain regions in a variety of chronic pain conditions [19,20,21,22,23]. The objective of the present study was to identify potential WM changes by analysing TBSS and ROI-restricted TBSS data from DTI scans of patients with MOH, a healthy control group (HC), and a group of MOH patients after drug withdrawal (MOH-DW).

## 2. Materials and Methods

### 2.1. Participants

This study included 23 patients aged 18–60 years who were admitted to the Headache Clinic of the Neurology Department of Ondokuz Mayıs University Faculty of Medicine, Samsun, Türkiye and diagnosed with MOH according to the International Headache Society (IHS) 2018 ICHD 3rd edition criteria [24]. Additionally, a control group of 23 demographically matched healthy individuals was included. All 23 patients and 23 healthy controls underwent baseline MRI; 16 patients and 16 controls completed the longitudinal protocol and underwent follow-up MRI, while the remaining seven patients did not complete follow-up (one excluded due to pregnancy, four discontinuing follow-up, and two excluded for technical issues).

All participants were thoroughly informed about the study, assured that their personal data would remain confidential, and provided written informed consent to participate in the study. A comprehensive medical history was obtained from the patients, followed by neurological and physical examinations. The Hamilton Depression Rating Scale was administered, and an MRI was performed prior to treatment.

Following withdrawal of the overused medication, patients received prophylactic treatment and, when required, rescue headache medication from an alternative pharmacological class. Each patient maintained a headache diary, which was reviewed during monthly outpatient visits. The diary recorded pain frequency, duration, intensity, characteristics, associated factors, and the frequency and dosage of analgesics used. Subjective headache pain intensity was assessed using a 0–10 visual analogue scale (VAS), with 0 indicating no pain and 10 indicating the worst imaginable pain.

Treatment response was classified as complete if pain frequency and severity were reduced by more than 50% and partial if the reduction was less than 50%. After clinical improvement, patients underwent a follow-up MRI examination; patients assessed at this time point are hereafter referred to as MOH-DW. The timing of the follow-up examination was not standardized across participants, and the interval between the baseline and follow-up MRI examinations was recorded in days. In one patient, sustained treatment response took longer to achieve, requiring extension of prophylactic therapy before the >50% improvement criterion was met; this patient’s follow-up MRI was accordingly performed later than in the remaining patients, contributing to the wide observed range. Of the 23 healthy controls originally enrolled, 16 individuals matched to the final MOH sample in age and sex were retained for analysis.

The HC group consisted of individuals who reported no history of primary headache or any other chronic or neuropsychiatric disorders.

Approval for this study was granted by the Ondokuz Mayıs University Faculty of Medicine Clinical Research Ethics Committee (reference number B.30.2.ODM.0.20.08/186-295-696-1424), under decision number OMÜ KAEK 2016/129. The thesis project (PYO.TIP.1904.16.009) received approval from the Ondokuz Mayıs University Scientific Research Projects Coordination Unit, and the necessary financial budget was allocated.

### 2.2. MRI Acquisition

The magnetic resonance imaging data were collected at the Department of Radiology, Ondokuz Mayıs University using a 3-Tesla magnetic resonance imaging scanner (Ingenia, Philips Healthcare, Best, The Netherlands) with a 16-channel head coil. Diffusion-weighted images were obtained using a single-shot echo planar imaging sequence with the following parameters: TR: 4286 ms, TE: 99 ms, FOV: 224 × 224 mm^2^, voxel size: 2 × 2 × 2 mm^3^ (no gap), matrix: 128 × 128. Each volume contained 60 straight axial slices covering the whole brain. In addition to b = 0 s/mm^2^ image, diffusion-weighted data were acquired in 32 directions at b = 800 s/mm^2^.

### 2.3. Diffusion-Weighted MRI Data Analysis

Diffusion weighted MRI data were analyzed using FSL software (FMRIB Software Library, version 6.0.3; Wellcome Centre for Integrative Neuroimaging, University of Oxford, Oxford, UK) [25].

The preprocessing pipeline included the following steps: (1) non-brain tissue was removed from the non-diffusion-weighted (b = 0) images with BET (Brain Extraction Tool); (2) eddy current-induced distortions and subjects’ head motion were corrected using eddy in FMRIB’s Diffusion Toolbox (FDT); (3) fractional anisotropy (FA), mean diffusivity (MD) and other tensor-derived metrics were generated by fitting a tensor model to the eddy-corrected diffusion data using the dtifit function in FDT; (4) radial diffusivity (RD) and axial diffusivity (AD) maps were computed using the diffusion tensor eigenvalues [26,27].

Whole-brain TBSS analysis was performed. FA data from all subjects were aligned into 1 × 1 × 1 mm standard Montreal Neurological Institute (MNI) space using the non-linear registration tool (FNIRT) [20,25]. A mean FA image was generated and thinned to create a mean FA skeleton representing the centers of white matter tracts common to all participants. The mean FA skeleton was thresholded at FA > 0.2, and individual FA images were projected onto this skeleton. MD, AD, and RD images were also transformed and projected onto the mean FA skeleton using the standard TBSS non-FA pipeline.

Voxel-wise statistical analyses were performed using non-parametric permutation testing implemented in FSL’s randomise, with 10,000 permutations and threshold-free cluster enhancement (TFCE) [28,29]. Statistical significance was defined as family-wise error (FWE)-corrected *p* < 0.05.

Two-sample unpaired *t*-tests were used to compare DTI metrics between healthy controls and patients with medication-overuse headache at the pre-withdrawal and post-withdrawal assessments, with age and sex included as covariates in these between-group analyses. To examine within-subject longitudinal differences, the pre- and post-withdrawal scans were analyzed using a paired repeated-measures general linear model implemented in FSL’s randomise. Both observations from each participant were entered into the same model; participant-specific regressors were included in the design matrix to account for individual-level effects, and the pre- and post-withdrawal observations from each participant were assigned to the same exchangeability block, supplied to randomise using the -e option, thereby restricting the permutation scheme according to the paired structure of the data and preventing repeated observations from being treated as independent. Two directional contrasts were specified (pre-withdrawal > post-withdrawal and post-withdrawal > pre-withdrawal). The interval between the baseline and follow-up MRI examinations, expressed in days, and treatment-related information were represented in the longitudinal model. Treatment-related information was entered as a categorical variable representing the specific prophylactic regimen received during the inter-scan interval, rather than as a continuous or ordinal dose-based score, because the regimens combined agents from different pharmacological classes for which no validated dose-equivalence framework relating to white-matter microstructure exists. Statistical inference used 10,000 permutations with two-dimensional threshold-free cluster enhancement (-T2), with significance defined as FWE-corrected *p* < 0.05 [28,29]. The longitudinal analysis was a complete-case analysis including exactly the same 16 patients at both time points; patients without a follow-up MRI were excluded, and no imputation was performed for missing follow-up data.

**Figure 1:**
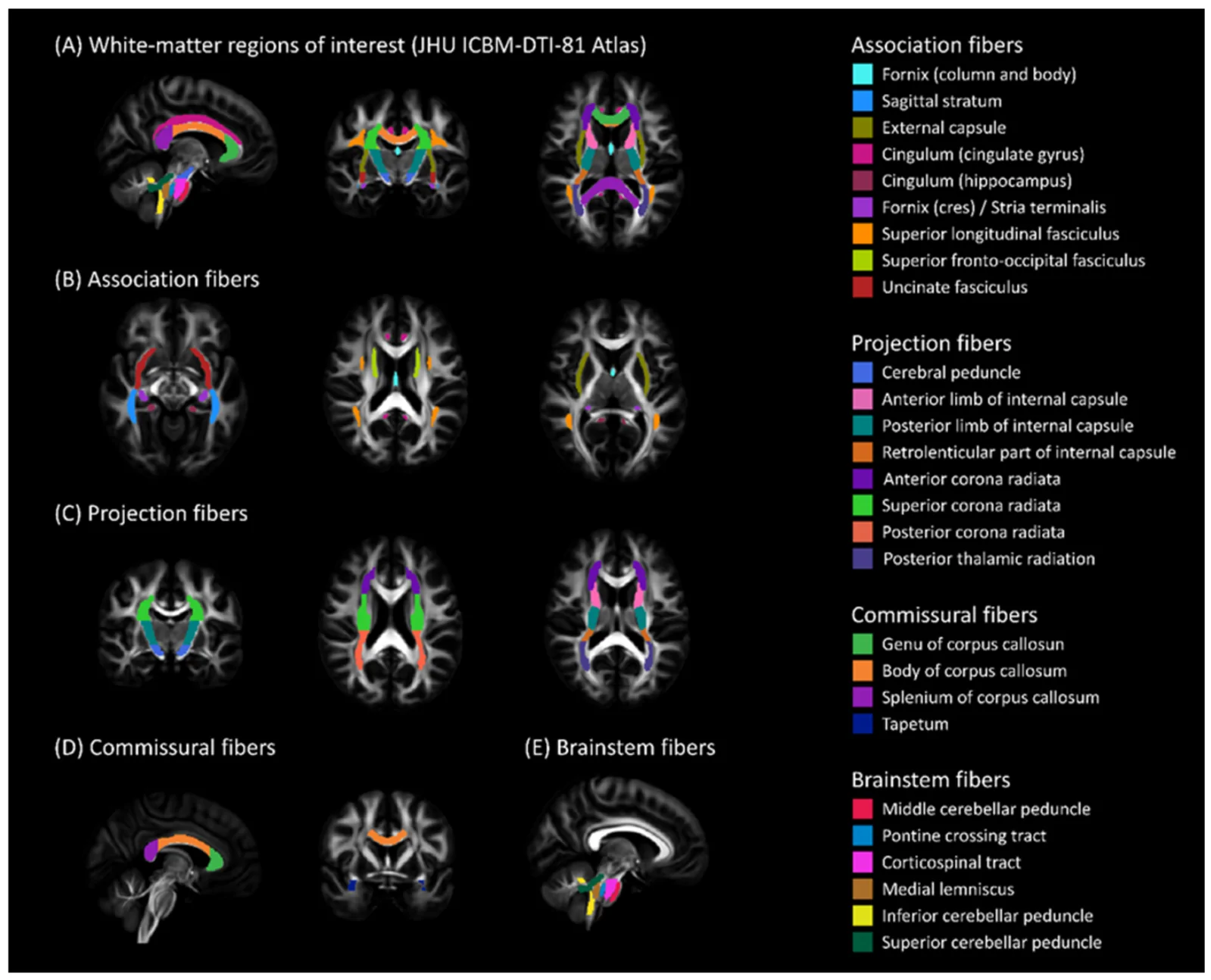
White-matter regions of interest (ROIs) used in the ROI-restricted tract-based spatial statistics (TBSS) analysis. White-matter tracts from the Johns Hopkins University ICBM-DTI-81 atlas are shown superimposed on a standard-space (FSL_HCP1065_FA_1mm) fractional anisotropy (FA) template. ROIs are grouped by fiber class: (**A**) all analyzed regions displayed in the sagittal, coronal, and axial planes; (**B**) association fibers; (**C**) projection fibers; (**D**) commissural fibers; and (**E**) brainstem fibers. Left and right homologous tracts are assigned the same color. The color of each individual tract is defined in the legend, which is organized into the four fiber classes (brainstem, projection, association, and commissural). Abbreviations follow the atlas nomenclature.

In addition to the whole-brain TBSS analysis, exploratory atlas-based, tract-specific voxel-wise analyses were performed to investigate spatially localized white matter alterations within anatomically defined white matter regions. For this purpose, ROI-restricted TBSS analyses were conducted separately for the white matter regions defined in the Johns Hopkins University (JHU) ICBM-DTI-81 White Matter Labels Atlas (Figure 1). Each atlas-defined ROI was intersected with the mean FA skeleton to generate a tract-specific skeleton mask, following a previously described approach [30]. A total of 27 anatomically defined white matter ROI masks from the JHU ICBM-DTI-81 atlas were examined separately. Although the ROI masks were defined before statistical testing based on the atlas, they were not selected on the basis of specific tract-level a priori hypotheses; therefore, the ROI-restricted analyses were considered exploratory. Within each ROI, voxel-wise statistical inference was performed using FSL’s randomise with 10,000 permutations and TFCE. Family-wise error correction was applied across voxels within each individual ROI, with statistical significance defined as FWE-corrected *p* < 0.05. No additional correction was applied across the set of 27 ROI analyses. FA was examined as the primary diffusion metric, and for ROIs showing significant FA differences, corresponding changes in MD, AD, and RD were subsequently investigated.

### 2.4. Statistical Analysis

Statistical analyses were performed using IBM SPSS Statistics, version 31.0 (Armonk, NY, USA). Age, sex and smoking status were compared between HC and MOH groups. Age was analyzed using the independent samples *t*-test. Gender distribution between the study groups was compared using Fisher’s exact test, while smoking status distribution was analyzed using Pearson’s chi-square test. The significance level was set at *p* < 0.05.

## 3. Results

### 3.1. Demographic and Clinical Results

One patient was excluded from the study due to pregnancy; four patients discontinued follow-up; and two patients were excluded due to technical issues. As a result, data from 16 MOH patients (15 female; mean age = 42.06 years, SD = 7.20) and 16 HC participants (15 female; mean age = 36.94 years, SD = 8.58) were analyzed. The groups were also matched in terms of smoking status. While 43.75% of the MOH patients were smokers, 37.5% of the healthy controls were smokers, and the difference was not statistically significant (χ^2^ = 0.13, *p* = 0.719) (Table 1).

Migraine without aura was the most frequent primary headache type, and simple analgesic overuse was slightly more common than combination analgesic overuse. Detailed clinical characteristics of the MOH group are presented in Table 2.

Clinical headache-diary measures indicated substantial clinical improvement at follow-up. The number of headache days decreased from a mean of 24.69 days (SD = 4.27; median = 25; range = 20–30) at baseline to 6.13 days (SD = 2.19; median = 5; range = 3–10) at follow-up. The mean within-patient reduction was 18.56 headache days (SD = 4.50; median = 20; range = 10–25). Reported monthly acute medication intake also decreased, from a mean of 29.84 (SD = 9.85; median = 22.5; range = 20–60) at baseline to 6.81 (SD = 2.69; median = 6; range = 3–12) at follow-up. Pain-intensity values decreased from a recorded baseline range of VAS 7–10 to VAS 1–4 at follow-up.

Following the discontinuation of the overused medication in the MOH group, rescue medication and prophylactic treatment were initiated from an alternative drug class. Details on prophylactic treatment are presented in Table 3.

### 3.2. TBSS Results

The whole-brain TBSS analysis revealed no statistically significant differences between the groups for any tensor metric (*p* > 0.05).

**Figure 2:**
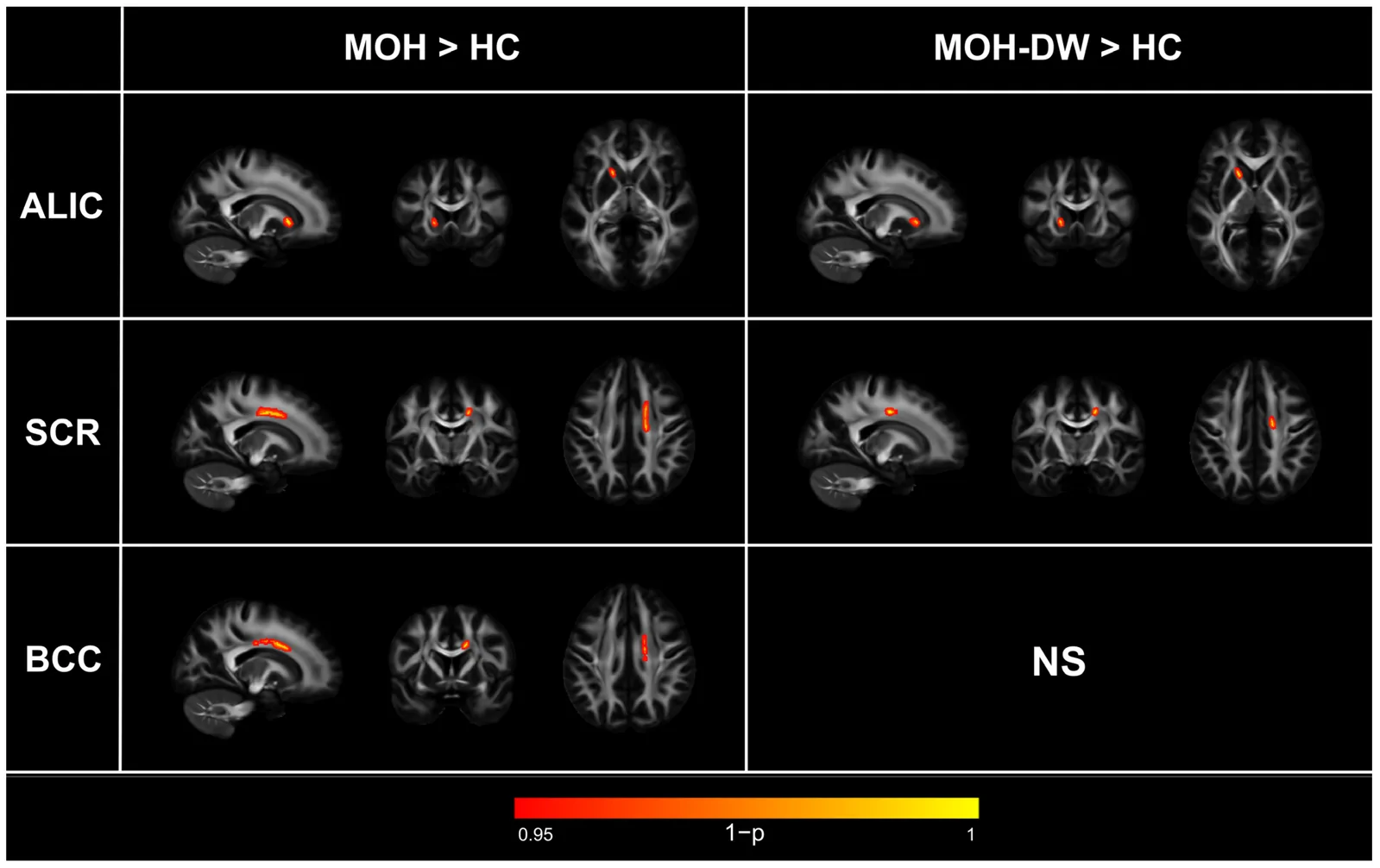
Tract-based spatial statistics (TBSS) comparison of fractional anisotropy (FA) between groups. Rows correspond to the three white-matter regions of interest: the anterior limb of the internal capsule (ALIC), superior corona radiata (SCR), and body of the corpus callosum (BCC). The left panel (MOH > HC) shows clusters of significantly higher FA in patients with medication overuse headache (MOH) than in healthy controls (HC); the right panel (MOH-DW > HC) shows the corresponding comparison between MOH patients after drug withdrawal (MOH-DW) and HC. For each comparison, representative sagittal, coronal, and axial sections are displayed in standard (MNI) space. Significant voxels (family-wise error [FWE]-corrected *p* < 0.05) are color-coded by 1 − *p*, with brighter colors indicating greater statistical significance. No significant difference (NS) was found in the BCC for the MOH-DW > HC comparison.

ROI-restricted TBSS analysis revealed statistically significant FA differences between the MOH and HC groups in the left anterior limb of the internal capsule (ALIC), the body of the corpus callosum (BCC), and the right superior corona radiata (SCR), with higher FA values in the MOH group. The direct within-subject comparison between pre- and post-withdrawal FA values did not reach statistical significance in any region. At the follow-up assessment, FA remained significantly higher than in HC in the left ALIC and right SCR (Table 4), whereas the difference between the MOH-DW and HC groups was not statistically significant in the BCC (Figure 2)—a pattern that, in the absence of a significant direct longitudinal change, may be interpreted as the disappearance of a detectable group difference rather than a confirmed reversal within patients.

### 3.3. Comparison of Other Tensor Metrics

In the MOH group, compared with the HC group, higher AD values were found in the left ALIC, BCC, and right SCR, whereas higher MD values were found only in the left ALIC. In the comparisons between the MOH-DW and HC groups, an increase in AD was observed only in the right SCR in the MOH-DW group (Table 5).

## 4. Discussion

Structural and functional alterations have been demonstrated in migraine. However, few studies focused on abnormalities in WM integrity, particularly in the CC [31,32,33]. In addition to its numerous other functions, the CC has been shown to play a role in the perception of pain, and there is evidence that its integrity is altered in situations accompanied by pain [34,35].

Szabó et al. reported that high FA values were observed in the left parieto-occipital WM in migraine patients with aura compared to the healthy control group. When these patients were compared with the patients with migraine without aura, high FA was found in the CC in migraine patients with aura [36].

Interestingly, the direction of the FA changes observed in our cohort differs from what has been reported in the most directly comparable study. Shibata et al., using a similar TBSS approach in a larger sample of migraine and MOH patients, found that FA in the corpus callosum was significantly lower in patients with medication overuse compared with healthy controls, a reduction generally interpreted as reflecting structural white matter injury from chronic overuse of analgesics and triptans [33]. In contrast, our ROI-restricted analysis showed higher FA values in the MOH group relative to HC in the BCC, the left ALIC and the right SCR. This discrepancy may partly reflect methodological differences, including field strength (1.5-T versus our 3-T acquisition), sample composition, or the stage of disease at the time of scanning, but it may also point to a genuinely different underlying microstructural process.

It should be noted that the group differences discussed in the following paragraphs were detected only in the ROI-restricted analyses; the whole-brain TBSS analysis did not reveal statistically significant differences between groups for any diffusion metric, and these findings should accordingly be interpreted as exploratory rather than confirmatory.

Elevated FA is not intrinsically incompatible with a pathological process. While a decrease in FA is the most frequently reported abnormality in chronic pain conditions and is generally attributed to demyelination or axonal loss, increased FA has also been described in several chronic pain states and has been proposed to reflect either compensatory neuroplastic reorganization or a reversible, edema-related process rather than fixed tissue damage. For example, patients with irritable bowel syndrome, another chronic visceral pain condition, have been shown to have higher FA in the CC and frontal WM alongside lower FA in thalamic and sensorimotor regions, a pattern attributed to region-specific neural reorganization rather than uniform degeneration [37]. Similarly, transient increases in FA have been documented in experimental models of reversible vasogenic edema, in which FA rises during the acute phase of blood-brain barrier disruption and returns to baseline once the underlying edema resolves [38]. A further possibility is activity-dependent myelination, whereby repeated nociceptive traffic along these pathways promotes oligodendrocyte activation and increased myelin deposition, a mechanism well described for other forms of repetitive neural activity and one that would likewise be expected to increase rather than decrease FA [39]. Within this framework, the absence of a statistically detectable BCC FA difference at follow-up may be compatible with an activity- or edema-related microstructural process rather than a fixed structural lesion. However, because the direct within-subject comparison was not statistically significant and withdrawal occurred concurrently with the introduction of alternative treatment, this remains a possible interpretation rather than a demonstrated treatment- or withdrawal-related change.

FA is a non-specific diffusion metric, with its value influenced by fiber organization, crossing-fiber geometry, axonal packing, myelination, and extracellular water content, such that an increase in FA does not necessarily correspond to a single specific biological process. Accordingly, the mechanistic explanations discussed above—compensatory neuroplasticity, reversible edema, activity-dependent myelination, and altered fiber orientation dispersion—remain plausible but not yet directly tested hypotheses; multimodal approaches such as NODDI or multi-shell diffusion acquisitions would be needed in future work to adjudicate between them.

The additional tensor metrics support this interpretation. AD, rather than RD, was the metric most consistently elevated alongside FA in the MOH group, with significantly higher AD in the left ALIC, the BCC and the right SCR, while MD was elevated only in the left ALIC (Table 4). An FA increase driven predominantly by AD, without a corresponding change in RD, is precisely the pattern reported in the reversible vasogenic edema model discussed above, strengthening the possibility that the WM changes seen here reflect a reversible, water-content-related process rather than fixed axonal or myelin damage [38]. This interpretation is also compatible with reports linking increased AD to pain and hyperalgesia in other chronic pain populations, such as Gulf War illness, where increased AD in prefrontal-projecting white matter tracts correlated with symptom severity [40]. An important caveat, however, is that AD and FA are both highly sensitive to crossing-fiber geometry: because most white matter voxels, including the internal capsule and corona radiata, contain multiple fiber populations, an apparent increase in AD and FA can also arise from disproportionate change in one fiber population relative to another rather than from a uniform increase in axonal coherence, so these metrics should not be interpreted as direct markers of tissue integrity in isolation [41]. This heterogeneity is not unique to our cohort: a recent systematic review of DTI imaging studies in migraine identified the CC, thalamic radiations and corona radiata as the WM regions most consistently reported to show diffusion abnormalities, while also cautioning that crossing-fiber contamination and other methodological factors likely contribute to the inconsistent direction of these findings across studies [42]. Notably, at the follow-up assessment, AD remained significantly higher than in HC only in the right SCR, mirroring the between-group pattern observed for FA; among the three regions studied, the group difference in the BCC was the one no longer statistically detectable at follow-up, whereas the corona radiata alteration appeared comparatively more stable.

A complementary explanation comes from neurite orientation dispersion and density imaging (NODDI), an extension of DTI that decomposes FA into a neurite density index (intracellular volume fraction) and an orientation dispersion index (ODI). In a NODDI study of patients with migraine and MOH from the same research group as reference-33, MOH was associated with reduced ODI, rather than reduced neurite density, specifically in the BCC, and ODI in this region was negatively correlated with attack frequency [43]. Since a reduction in fiber orientation dispersion, that is, a more parallel, less crossing fiber geometry, can itself elevate conventional DTI metrics such as FA and AD without any change in axonal density, this raises the possibility that the FA and AD elevations we observed in the BCC reflect altered fiber coherence rather than either injury or straightforward edema. Combining conventional DTI with NODDI to disentangle these possibilities is an approach only very recently applied to primary headache disorders: a 2026 study using both DTI and NODDI in chronic tension-type headache (CTTH) similarly reported diffusivity changes across multiple pain-processing and association-fiber regions, supporting the view that multi-compartment diffusion models are becoming an important complement to conventional DTI metrics across different headache phenotypes, even though that particular study examined CTTH rather than MOH specifically, and in fact excluded patients with medication overuse; the two entities are nonetheless not mutually exclusive, since medication overuse can develop on top of a primary tension-type headache, as was the case for three patients in our MOH cohort, who had tension-type headache as their underlying primary headache diagnosis [44].

The ALIC warrants specific comment, as it was among the most consistently altered regions in our cohort and, together with the SCR, one of only two regions in which FA and AD elevations persisted after drug withdrawal. Anatomically, the ALIC carries the anterior thalamic radiation, connecting the mediodorsal and anterior thalamic nuclei to the prefrontal cortex; this thalamocortical relay is thought to support the affective-cognitive, rather than purely sensory-discriminative dimension of pain processing, which may help explain why this tract showed a more persistent signature than the CC. Diffusion changes in the internal capsule have also been reported in other headache-associated conditions, generally in the opposite direction to our findings: in spontaneous intracranial hypotension, FA and AD in the ALIC were decreased while RD was increased, a pattern attributed to traction- or pressure-related axonal injury [45]. This contrast with an injury-type diffusion profile further supports the view that the changes we observed in MOH reflect a distinct, and at least partially reversible, process rather than a pattern of WM damage common to all headache-related conditions. The prefrontal cortex is also among the targets of cognitive behavioral therapy or neuromodulation in headache patients.

An alternative, and not mutually exclusive, explanation for the white-matter changes observed after withdrawal is a direct pharmacological or neuroplastic effect of the prophylactic medications themselves, rather than an effect of withdrawal per se. This possibility has direct precedent in the literature: in two randomized, placebo-controlled trials in persistent depressive disorder, treatment with the SNRIs duloxetine and desvenlafaxine altered diffusion tensor metrics in several white-matter regions, including the corona radiata, normalizing fractional anisotropy toward values seen in healthy controls, whereas diffusion metrics in placebo-treated patients deviated further from normal [46]. Because an SNRI was part of the prophylactic regimen in two of our MOH-DW patients (Table 3), and because the corona radiata was one of the tracts showing a persistent FA elevation in our cohort, a direct antidepressant effect on this specific tract cannot be excluded. The inter-scan interval and treatment-related information were included as covariates in the longitudinal statistical model (Section 2.4), but the limited sample size did not allow formal adjustment for the specific prophylactic regimen or drug class. Valproic acid, used as monotherapy or in combination in ten of our sixteen patients, is a histone deacetylase inhibitor shown to enhance oligodendrocyte survival, promote oligodendrocyte progenitor differentiation, and increase myelinated axonal density after experimental stroke in rodents [47]. Although this evidence comes from an animal injury model rather than the clinical population and dosing used here, it establishes that valproic acid is pharmacologically capable of modulating white-matter and myelin-related processes, adding biological plausibility to a direct drug effect. Topiramate and propranolol, the remaining prophylactic agents used in our cohort, likewise have documented central nervous system effects distinct from analgesic withdrawal. Topiramate reduces prefrontal cortical activation during language tasks on functional MRI in patients with epilepsy [48]. Propranolol crosses the blood–brain barrier and modulates prefrontal–hippocampal circuits involved in fear-memory extinction [49]. To our knowledge, however, neither agent’s effect on white-matter diffusion metrics has been directly studied. Taken together, these considerations indicate that the white-matter changes observed at follow-up may not be attributable solely to withdrawal of the overused medication, and that a pharmacological effect of the prophylactic regimen is a biologically plausible contributing factor that the present study was not designed to isolate.

A further limitation relates to the clinical heterogeneity of the underlying primary headache disorder within the MOH group, which included patients with migraine without aura (*n* = 11), migraine with aura (*n* = 2), and tension-type headache (*n* = 3) (Table 2). Because migraine and tension-type headache are thought to differ in their underlying pathophysiology, including the extent of trigeminovascular and central sensitization, it is possible that the reported white-matter alterations partly reflect the specific mix of primary headache phenotypes in our cohort rather than a feature of MOH uniformly. Given the small subgroup sizes, we were not able to formally test for a phenotype-specific effect on diffusion metrics, and this heterogeneity should be considered when generalizing our findings; future studies in phenotypically homogeneous MOH cohorts are needed.

Autonomic activity plays a role in migraine attacks, and because autonomic control is predominantly mediated by the right hemisphere, microstructural changes in right hemisphere pathways can be expected in chronic headache patients [50]. For this reason, it is possible that we may have identified different microstructural changes in the right SCR in the patient and treatment groups in comparison to the healthy group. Nevertheless, further studies are required to establish a correlation between autonomic dysfunction and imaging findings.

A useful conceptual framework for these findings is the transition from adaptive to maladaptive plasticity across the migraine spectrum. Repeated trigeminovascular activation and thalamocortical hyperexcitability are thought to drive activity-dependent white matter remodelling, which in the episodic phase may represent an adaptive, compensatory response [51]. In the setting of medication overuse, this same process may become maladaptive, contributing to central sensitization and persistent network dysfunction [52]. Consistent with this view, an emerging body of work reframes migraine not as a degenerative white matter disease but as a dynamic process of network reorganization, a perspective that fits well with the increased and partially reversible diffusion changes observed here [53].

From a clinical perspective, the presence of FA differences in the ALIC and SCR at follow-up, in contrast to the absence of a statistically detectable difference in the BCC, raises the possibility that the diffusion patterns observed across these regions may differ over time. However, these findings cannot be attributed exclusively to withdrawal of the overused medication, because prophylactic and alternative rescue treatments were introduced during the same interval. If confirmed in larger, longitudinal cohorts, ROI-based FA measurements in these tracts could serve as an adjunctive imaging marker of treatment response, complementing clinical follow-up based on headache diaries alone.

This study has several limitations. First, although 23 patients were initially recruited, the final longitudinal analysis included 16 patients, corresponding to an attrition and exclusion rate of approximately 30% (one patient excluded due to pregnancy, four discontinuing follow-up, and two excluded for technical issues); the healthy control group was correspondingly limited to 16 individuals matched in age and sex. The resulting modest sample size limited statistical power, reduced the sensitivity of the whole-brain TBSS analysis to potentially small or spatially distributed effects, and increased the risk of type II error; the absence of statistically significant whole-brain findings should therefore not be interpreted as evidence for the absence of white matter microstructural alterations. At the same time, the modest sample size increases susceptibility to Type I error and sample-specific variability, such that some of the significant ROI-restricted findings detected in this small cohort may not be robust or reproducible in an independent sample. The predominantly female sample also precludes conclusions about sex-related differences, and the loss of nearly 30% of enrolled patients raises the possibility of selection bias, as we were not able to formally compare baseline characteristics between completers and non-completers. Second, only the ROI-restricted analyses, not whole-brain TBSS, detected group differences. Although TFCE-based FWE correction was applied across voxels within each individual ROI, no additional correction was applied across the complete set of 27 atlas-defined ROI analyses; consequently, the overall Type I error rate across the ROI analyses was not controlled, and the possibility of false-positive findings due to ROI-level multiplicity cannot be excluded. This limitation is particularly important given the absence of significant whole-brain findings; accordingly, the ROI-restricted results are exploratory and hypothesis-generating in nature rather than confirmatory. Third, the direct within-subject comparison between pre- and post-withdrawal DTI metrics, modeled as a paired repeated-measures design in FSL’s randomise, did not reach statistical significance in any region; the loss of a significant MOH-DW-versus-HC difference in the CC therefore reflects the disappearance of a detectable group difference rather than confirmed within-subject reversibility. Fourth, all patients received heterogeneous prophylactic treatment after withdrawal (Table 3), and its potential independent effect on white-matter microstructure could not be statistically disentangled from that of medication withdrawal itself, despite the inclusion of treatment-related information as a covariate. Fifth, the timing of the follow-up MRI examination was not standardized, and the interval between the baseline and follow-up MRI examinations varied considerably across participants (mean ± SD, 147.75 ± 90.12 days; median, 132 days; range, 91–476 days). Although the inter-scan interval was represented in the longitudinal statistical model, this adjustment may not have fully accounted for differences in the stage of clinical or microstructural change at follow-up, particularly given the modest sample size; this temporal variability may have reduced sensitivity to longitudinal effects and limits interpretation of their temporal course. Sixth, the MOH group included patients with different underlying primary headache disorders (migraine without and with aura, and tension-type headache), and small subgroup sizes precluded testing for a phenotype-specific effect. Seventh, we did not formally test associations between clinical measures of treatment response—headache frequency, headache days per month, acute medication use, pain intensity, and disability—and the observed diffusion changes, particularly for the CC finding; these correlations were not performed given the sample size and the multiplicity already introduced by the 27 ROI analyses, and should be addressed in future, adequately powered studies. Eighth, standardized effect sizes and confidence intervals were not reported for the principal comparisons: because these analyses were voxel-wise within the TBSS skeleton, with non-identical sets of significant voxels across comparisons, a single tract-averaged effect size would not represent the voxel-wise effects tested, and deriving effect sizes from the significant clusters themselves would introduce circular, selection-related inflation; we instead report peak test statistics, cluster extent, peak MNI coordinates, and TFCE-based FWE-corrected *p*-values for each finding. Finally, the availability of only a single post-withdrawal MRI time point precludes conclusions about the longer-term trajectory of these changes, and the absence of NODDI acquisition means we cannot determine whether the observed FA and AD elevations reflect altered neurite density, reduced orientation dispersion, or both; future studies with larger samples, standardized prophylactic treatment, multiple post-withdrawal time points, and multi-shell diffusion acquisitions should address these gaps.

## 5. Conclusions

Taken together, our results add to a small but growing body of DTI literature suggesting that WM microstructure in MOH is dynamic rather than fixed, consistent with a process of network reorganization rather than fixed injury. The absence of a statistically significant MOH-DW versus HC difference in the BCC at follow-up, together with the persistence of FA and AD differences in the ALIC and SCR—tracts implicated in the affective-cognitive rather than purely sensory dimension of pain—raises the hypothesis that the diffusion patterns observed across these regions may differ over time. However, because the direct within-subject comparison was not statistically significant and medication withdrawal occurred concurrently with the introduction of alternative treatment, these observations should not be interpreted as evidence of a specific withdrawal effect or a demonstrated trajectory of recovery. These WM alterations may represent both markers and modifiers of migraine-related pathology, and it remains unclear whether they are a cause or a consequence of chronic medication overuse. Future longitudinal studies with larger samples, multiple post-withdrawal imaging time points, and complementary diffusion metrics such as mean, axial and radial diffusivity will be needed to clarify whether the FA elevations observed here reflect adaptive plasticity, subclinical edema, reduced fiber orientation dispersion or an early, potentially maladaptive stage of a process that becomes degenerative with more prolonged medication overuse.

## Figures and Tables

**Table 1:** Demographic and clinical characteristics of study groups.

Characteristic	MOH (*n* = 16)	HC (*n* = 16)	Test Statistic	*p*
Age, years—mean (SD)	42.06 (7.20)	36.94 (8.58)	*t*(30) = 1.83	0.077 ^a^
Female sex—*n* (%)	15 (93.75)	15 (93.75)	-	1.000 ^b^
Smoker—*n* (%)	7 (43.75)	6 (37.50)	χ^2^(1) = 0.13	0.719 ^c^

^a^ Independent-samples *t*-test; ^b^ Fisher’s exact test; ^c^ Pearson’s chi-square test. Data are presented as mean (SD) or *n* (%). SD = Standard Deviation; MOH = Medication Overuse Headache Patients before drug withdrawal; HC = Healthy Control.

**Table 2:** Clinical characteristics of the MOH group.

Characteristic	MOH (*n* = 16)
**Primary headache type—*n* (%)**	
Migraine without aura	11 (68.75)
Migraine with aura	2 (12.50)
Tension-type headache	3 (18.75)
**Type of medication overuse—*n* (%)**	
Simple analgesic	9 (56.25)
Combination analgesic	7 (43.75)
Duration of primary headache, years—mean (SD)	14.6 (5.6)
Duration of medication overuse, years—mean (SD)	3.6 (1.4)
Interval between baseline and follow-up MRI examinations, days—mean (SD); median; range ^a^	147.75 (90.12); 132; 91–476

^a^ Time elapsed between the baseline and follow-up MRI examinations. Data are presented as *n* (%) or mean (SD). SD = Standard Deviation; MRI = Magnetic Resonance Imaging.

**Table 3:** Prophylactic treatment in the MOH group.

Prophylactic Regimen	*n* (%)
**Monotherapy**	**10 (62.5)**
Valproic acid	4 (25.0)
Topiramate	3 (18.8)
Propranolol	2 (12.5)
Flunarizine	1 (6.3)
**Combination therapy**	**6 (37.5)**
Valproic acid + propranolol	1 (6.3)
Valproic acid + SNRI	1 (6.3)
Propranolol + topiramate	1 (6.3)
Propranolol + amitriptyline	2 (12.5)
Propranolol + flunarizine	1 (6.3)
**Total**	**16 (100)**

Data are presented as *n* (%). SNRI = Serotonin–Norepinephrine Reuptake Inhibitor.

**Table 4:** ROI-restricted TBSS analysis results for fractional anisotropy (FA).

Comparison	Region	Cluster Size (Voxels)	Peak MNI Coordinate (mm)			*t*	*p*
			*x*	*y*	*z*		
**MOH > HC**	Anterior limb of the internal capsule (L)	47	−17	18	−1	5.03	0.004
	Body of the corpus callosum	110	15	4	32	3.74	0.028
	Superior corona radiata (R)	150	17	−8	37	4.00	0.010
**MOH-DW > HC**	Anterior limb of the internal capsule (L)	49	−18	17	2	4.09	0.007
	Superior corona radiata (R)	26	18	−9	39	3.77	0.039

MOH > HC and MOH-DW > HC indicate clusters with significantly higher FA in the patient group than in controls. Cluster size denotes the number of significant voxels. Coordinates are reported in MNI space (mm). *p*-values were corrected for multiple comparisons using the family-wise error (FWE) rate, with significance set at *p* < 0.05. ROI = Region of Interest; TBSS = Tract-Based Spatial Statistics; FA = Fractional Anisotropy; MNI = Montreal Neurological Institute; L = Left; R = Right; HC = Healthy Control; MOH = Medication Overuse Headache Patients before drug withdrawal; MOH-DW = Medication Overuse Headache Patients after drug withdrawal.

**Table 5:** ROI-restricted TBSS analysis results for axial diffusivity (AD) and mean diffusivity (MD).

Comparison	Metric	Region	Cluster Size (Voxels)	Peak MNI Coordinate (mm)			*t*	*p*
				*x*	*y*	*z*		
**MOH > HC**	**AD**	Anterior limb of the internal capsule (L)	65	−17	18	0	5.08	<0.001
		Body of the corpus callosum	21	16	−13	35	4.09	0.042
		Superior corona radiata (R)	147	18	−14	38	4.25	0.007
	**MD**	Anterior limb of the internal capsule (L)	26	−20	8	13	3.92	0.026
			10	−23	−6	18	4.72	0.033
**MOH-DW > HC**	**AD**	Superior corona radiata (R)	34	19	−11	40	3.91	0.036

MOH > HC and MOH-DW > HC indicate clusters with significantly higher values of the respective metric (AD or MD) in the patient group than in controls. Cluster size denotes the number of significant voxels. Coordinates are reported in MNI space (mm). *p*-values were corrected for multiple comparisons using the family-wise error (FWE) rate, with significance set at *p* < 0.05. ROI, region of interest; TBSS = Tract-Based Spatial Statistics; AD = Axial Diffusivity; MD = Mean Diffusivity; MNI = Montreal Neurological Institute; L = Left; R = Right; HC = Healthy Control; MOH = Medication Overuse Headache Patients before drug withdrawal; MOH-DW = Medication Overuse Headache Patients after drug withdrawal.

## Data Availability

The datasets from this study are present in the paper or can be obtained from the corresponding author upon request.
